# *In Vivo* Antihyperglycemic Activity of a Lanosteryl Triterpene from *Protorhus longifolia*

**DOI:** 10.3390/molecules200713374

**Published:** 2015-07-22

**Authors:** Rebamang A. Mosa, Nkosinathi D. Cele, Sihle E. Mabhida, Samkelisiwe C. Shabalala, Dambudzo Penduka, Andy R. Opoku

**Affiliations:** Department of Biochemistry and Microbiology, University of Zululand, Private Bag X1001, KwaDlangezwa 3886, South Africa; E-Mails: melucy.cele@gmail.com (N.D.C.); sihlemabhida@gmail.com (S.E.M.); samk2mac@gmail.com (S.C.S.); propafadzo@gmail.com (D.P.); opokuA@unizulu.ac.za (A.R.O.)

**Keywords:** triterpene, antihyperglycemic activity, antioxidant status

## Abstract

Control of postprandial hyperglycemia is crucial in the management of diabetes mellitus. Despite the use of the current hypoglycemic drugs, incidence of diabetes and related diseases continue to increase. This study aimed at evaluating the *in vivo* antihyperglycemic activity of methyl-3β-hydroxylanosta-9,24-dien-21-oate (**RA-3**), a lanosteryl triterpene isolated, and characterized from *Protorhus longifolia* stem bark. Spectroscopic data analysis was used to establish and verify the structure of the triterpene. The antihyperglycemic activity of the triterpene was evaluated in an STZ-induced diabetes rat model. The experimental animals were orally administered with **RA-3** (100 mg/kg body weight) daily for 14 days. An oral glucose tolerance test was also performed. The animals were euthanized and biochemical analysis of antioxidant status, some glycolytic enzymes and glycogen content were conducted on serum and liver samples, respectively. **RA-3** exhibited hypoglycemic activity by reducing blood glucose levels by 37%. The triterpene also improved glucose tolerance in the diabetic rats. Relatively higher hepatic glycogen content, hexokinase and glucokinase activity with a decrease in glucose-6-phosphatase activity were observed in the triterpene-treated diabetic group when compared with the diabetic control group. The triterpene treatment further increased antioxidant status of the diabetic animals; increased activity of superoxide dismutase and catalase were observed along with a decrease in malondialdehyde content. The results indicate potential pharmaceutical effects of lanosteryl triterpene in the management of diabetes mellitus.

## 1. Introduction

Diabetes mellitus, one of the metabolic disorders characterized by chronic hyperglycemia, is a serious global health concern rapidly reaching epidemic levels. Its continuously escalating prevalence is likely to reach 439 million people by 2030 [1]. Type 2 (an insulin-independent diabetes mellitus) diabetes is responsible for over 90% of all cases of diabetes [2]. Hyperglycemia resulting from defects in insulin secretion, insulin action, or both, is considered the main cause of the debilitating effects of diabetes [3]. The hyperglycemia and hyperlipidemia commonly observed in diabetic patients are considered the main contributors to the development of micro- and macro-vascular complications [3,4]. Experimental evidence has also indicated the role of oxidative stress in the pathogenesis and complications of diabetes [5]. Thus control of postprandial hyperglycemia, hyperlipidemia, and oxidative stress are crucial in the treatment of diabetes.

New drugs are continually being tested and new strategies developed to prevent and treat diabetes. Some of the current diabetic therapies include use of carbohydrate digestive enzyme inhibitors [6], thus limiting glucose intestinal absorption and the use of insulin, insulin mimetics [7], and insulin secretagogues [8] to enhance cellular glucose uptake. However, adverse effects such as gastrointestinal discomfort, nausea, and weight gain [6] associated with the current oral antidiabetic drugs have stimulated a search for new, safe, and effective agents.

There is a growing interest in the use of medicinal plants and plant-derived compounds as alternative potentially safe antidiabetic drugs. Several studies [4,9] support the potential use of plant-derived triterpenes as new hypoglycemic agents. Various multiple biological activities of plant-derived triterpenoids with apparent effects on glucose absorption, cellular glucose uptake, insulin secretion, and relief of diabetic complications have also been documented [8,10,11]. The *in vivo* hypolipidemic [12] and *in vitro* hypoglycemic [13] activities of the lanosteryl triterpene from *Protorhus longifolia* stem bark have been reported. Increased plasma lipid levels inhibit cellular glucose uptake and utilization, key to the development of insulin resistance and type 2 diabetes.

This work was aimed at investigating the hypoglycemic activity of the triterpene, methyl-3β-hydroxylanosta-9,24-dien-21-oate, from *P. longifolia in vivo*.

## 2. Results and Discussion

### 2.1. Compound Identification

**Figure 1:**
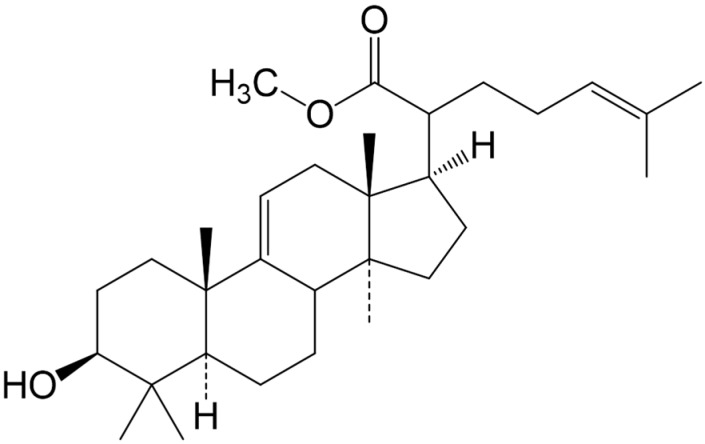
Methyl-3β-hydroxylanosta-9,24-dien-21-oate (**RA-3**).

The structure of the triterpene (Figure 1) was verified on the basis of spectral (^1^H- and ^13^C-NMR, IR) data analysis. The triterpene (Methyl-3β-hydroxylanosta-9,24-dien-21-oate, **RA-3**) was obtained as white crystals with estimated >95% purity. The physical and spectral data of the triterpene have previously been given and were all in agreement with the literature data [12,13].

### 2.2. Antihyperglycemic Activity

Chronic hyperglycemia is considered the main cause of the debilitating effects of diabetes mellitus [3]. Thus, control of postprandial hyperglycemia is crucial in the management of diabetes and its complications. Literature supports plant-derived triterpenoids as potential new antidiabetic agents [4,9,10,11].

**Figure 2:**
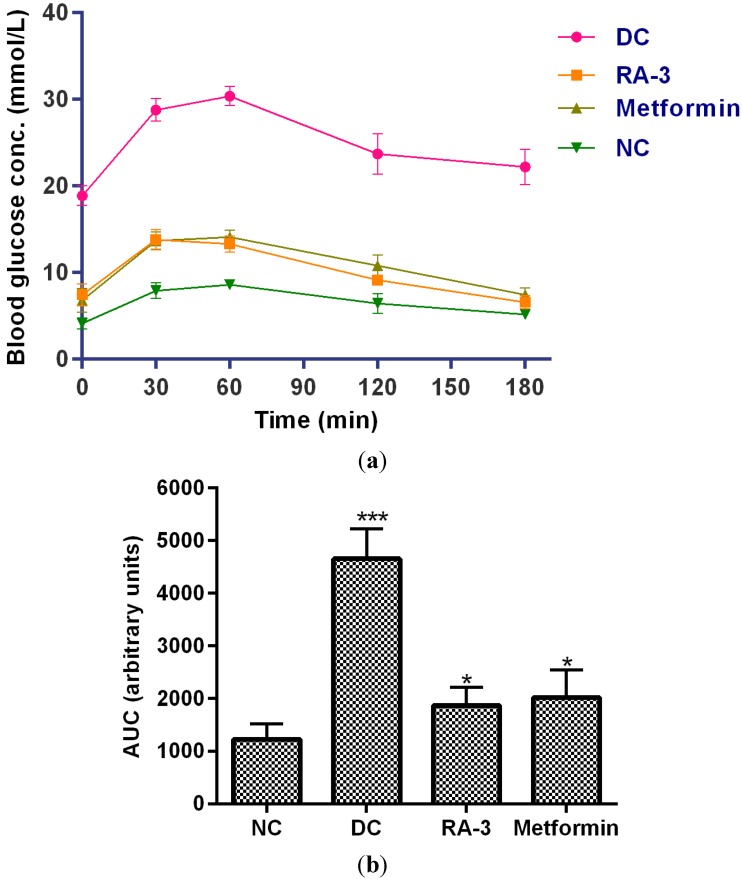
(**a**) Effect of **RA-3** on glucose tolerance in diabetic rats. The overnight fasted rats were orally given a glucose load (2 kg/kg b.w). Changes in blood glucose levels were monitored at 0, 30, 60, 90, 120 and 180 min. Data were expressed as mean ± SEM, (*n* = 5); **NC** = Non-diabetic control, **DC** = Diabetic control, **RA-3** = Diabetic + **RA-3**, **Metformin** = Diabetic + metformin; (**b**) AUCs of glucose tolerance test in rats after 14 days treatment with **RA-3** and metformin. Data were expressed as mean ± SEM, (*n* = 5); * *p* < 0.05, *** *p* < 0.001 *vs.* NC.

In this study, the *in vivo* hypoglycemic effect of the triterpene was evaluated in STZ-induced diabetic rats. The triterpene (100 mg/kg b.w) caused a significant reduction (37%) in blood glucose levels (Table 1) after 14 days of treatment, thus exhibiting hypoglycemic effect. The compound did not show any hypoglycemic effect in the non-diabetic animals. The hypoglycemic activity of the compound was further substantiated by its improvement of glucose tolerance in the diabetic rats (Figure 2a). The triterpene significantly decreased blood glucose levels in the oral glucose loaded diabetic rats within 3 h. The activity of the compound was comparable to that of metformin, a standard antidiabetic drug. An impaired glucose tolerance was observed in the diabetic control group animals. Compared to the larger area under the curve (4657.50 units) of the diabetic control group (Figure 2b), area under the curves (AUCs) of the triterpene (1870.95 units) and metformin (2019.35 units) treated diabetic groups was significantly decreased. Similar glycemic control has also been reported for other compounds including the *Syzigium aromaticum*-derived triterpenes; maslinic acid and oleanolic acid in STZ-induced diabetic rats following an oral glucose load [14]. The triterpene from *P. longifolia* has also recently been reported to have potent hypolipidemic effect [12] which is crucial in the prevention of diabetic complications.

**Table 1:** Effect of **RA-3** on blood glucose levels (mmol/L) of diabetic rats.

Group	Day 0	Day 7	Day 14	% Decrease
Non-diabetic control	5.40 ± 0.38	5.40 ± 0.26	5.40 ± 0.36	
Non-diabetic + **RA-3**	5.50 ± 0.25	5.40 ± 0.17	5.30 ± 0.17	
Diabetic + 2% T20	16.50 ± 3.09	14.00 ± 4.94	18.90 ± 3.29	
Diabetic + **RA-3**	11.80 ± 1.19	10.20 ± 4.50	7.45 ± 1.58 *	37
Diabetic + metformin	18.50 ± 2.12	6.75 ± 0.55 *	6.80 ± 1.60 *	63

Data were expressed as mean ± SEM, (*n* = 5); * *p* < 0.05 *vs.* diabetic control. T20 = Tween 20.

Decreased rate of glycolysis and blocked glycogenesis which both stimulate gluconeogenesis are some of the metabolic changes in diabetes mellitus. Plant-derived triterpenes are known to exert their hypoglycemic activity through various mechanisms which include stimulation of cellular glucose uptake and insulin secretion [10,11]. The ability of **RA-3** to stimulate cellular (C2C12 myocytes and 3T3-L1 adipocytes) glucose uptake has recently been reported [13]. In this study, effect of **RA-3** on some glycolytic enzymes (HK, GK, G6Pase) and glycogen synthesis was evaluated and the results are given in Table 2. The triterpene effectively increased the hepatic glycogen content, activity of HK and GK, while the G6Pase activity was decreased in the triterpene-treated diabetic group when compared to the diabetic control group. While the HK and GK activities in the triterpene-treated group were comparable to that in the normal control group, a marked inhibition of G6Pase activity was observed. This is supported by the relatively higher glycogen content observed in the triterpene-treated groups than in the normal control group. Similar activities of other triterpenes on the selected glycolytic enzymes and hepatic glycogen content in diabetic rats have been reported [15,16]. The observed activation of HK and GK along with inhibition of G6Pase, an enzyme that catalyzes the terminal step in both glycogenolysis and gluconeogenesis, further support the insulinogenic activity of the triterpene. Gutierrez [4] has also reported this insulinogenic character of some triterpenes from *Prosthechea michuacana* in STZ-induced diabetic rats.

**Table 2:** Effect of **RA-3** on hepatic glycogen content, HK, GK and G6Pase activity in the diabetic treated animals.

Group	HK (Units/mL)	GK (Units/mL)	G6Pase (Units/mL)	Glycogen Content (mg/g)
Non-diabetic control	0.73 ± 0.05 *	0.52 ± 0.01 *	1.42 ± 0.22	7.50 ± 0.09
Non-diabetic + **RA-3**	0.83 ± 0.00 *	0.68 ± 0.02 *	0.81 ± 0.05 *	8.00 ± 0.04
Diabetic + 2% T20	0.06 ± 0.01	0.02 ± 0.02	1.73 ± 0.22	6.00 ± 0.08
Diabetic + **RA-3**	0.74 ± 0.01 *	0.53 ± 0.02 *	0.99 ± 0.11 *	9.00 ± 0.18
Diabetic + metformin	0.93 ± 0.04 *	0.74 ± 0.01 *	1.53 ± 0.28	7.00 ± 0.00

Values are expressed as mean ± SEM (*n* = 5), * *p* < 0.05 *vs.* diabetic control. T20 = Tween 20.

Hyperglycemic-induced oxidative stress plays a central role in the development and complications of diabetes. Various other studies have demonstrated that plant-derived compounds including triterpenes significantly increase the endogenous antioxidant enzymes in diabetic animals [4,17,18,19]. The results on the effect of **RA-3** on MDA content, GSH content, SOD and CAT activity are presented in Table 3. The SOD and CAT activities were effectively increased along with decreased MDA contentinthe triterpene-treated diabetic animals compared to the diabetic control group. A relatively higher antioxidant status with lowered MDA (a product of lipid peroxidation) levels in the triterpene-treated diabetic rats indicates potential of **RA-3** to alleviate oxidative stress and subsequently prevent the development and progression of diabetes mellitus. The higher antioxidant effect observed in the metformin-treated diabetic animals relative to the diabetic control group is consistent with previous reports [20,21] on the antioxidant-like effect of this antidiabetic agent.

**Table 3:** Effect of **RA-3** on MDA level, GSH content, SOD and CAT activity.

Group	GSH (nmol/mL)	SOD (Units/mL)	CAT (Units/mL)	Antioxidant Status (mM)	MDA (nmol/µL)
ND control	22.31 ± 0.06 *	35.08 ± 0.04 *	40.10 ± 0.01 *	0.089 ± 0.12 *	0.40 ± 0.01
ND + **RA-3**	18.63 ± 0.10 *	32.90 ± 0.14 *	32.06 ± 0.22 *	0.135 ± 0.01 *	0.30 ± 0.04
D + 2% T20	13.50 ± 0.04	21.51 ± 2.41	13.31 ± 1.02	0.002 ± 0.29	0.90 ± 0.02
D + **RA-3**	13.32 ± 0.12	45.24 ± 1.07 *	30.12 ± 0.41 *	0.032 ± 0.04 *	0.40 ± 0.08
D + metformin	14.71 ± 0.10	41.71 ± 0.12 *	30.22 ± 0.19 *	0.199 ± 0.31 *	0.60 ± 0.03

Data are expressed as mean ± SEM, (*n* = 5). * *p* < 0.05 *vs.* diabetic control, D = diabetic, ND = non-diabetic.

## 3. Experimental Section

### 3.1. General

Unless otherwise stated, all chemicals, assay kits and reagents used were purchased from Sigma-Aldrich Chemical Co. (St. Louis, MO, USA).

The method described in Machaba *et al*. [12] was followed in the extraction and isolation of the triterpene from stem bark of *P. longifolia*. Briefly, the powdered plant material was sequentially extracted (1:5 *w*/*v*) with *n*-hexane and chloroform (Merck, Darmstadt, Germany). The triterpene was then isolated from the chloroform extract using column chromatography (24 × 700 mm; silica gel 60; 0.063–0.2 mm; 70–230 mesh ASTM, Merck), and eluted with a stepwise *n*-hexane–ethyl acetate solvent system (9:1 to 3: 7). Thin layer chromatography (silica gel 60 TLC aluminium sheets 20 cm × 20 cm, F_254_, Merck) was used to analyze the collected 20 mL fractions. The desired compound (**RA-3**) was obtained following recrystallization in ethyl acetate. The structure of the triterpene was established and confirmed through use of NMR (^1^H-^1^H, ^13^C-^13^C, in DMSO, Bruker 600 MHz, Bruker, Fremont, CA, USA), and IR (Perkin-Elmer 100 FTIR, Waltham, MA, USA) spectral data analysis. Melting point was also confirmed using melting point apparatus (Stuart SMP 11, Shalom Instruments supplies, Durban, South Africa).

### 3.2. Plant Material

The stem barks of *P. longifolia* were freshly harvested from Hlabisa, KwaZulu-Natal, SA. The plant (voucher specimen number RA01UZ) was verified by Dr. N.R. Ntuli from the Botany Department, University of Zululand, South Africa. The plant material was then air-dried and ground to powder.

### 3.3. Animals

Approval for use of experimental animals and procedures was obtained from the University of Zululand Research Ethics Committee (UZREC 171110–030 Dept. 2013/23). *Sprague-Dawely* rats, weighing 150–200 g were obtained from the Biochemistry and Microbiology Departmental animal house, University of Zululand. The animals were kept under standard conditions as outlined in the institutional guidelines for caring of experimental animals. The animals were allowed to acclimatize for five days with free access to enough rat feed and drinking water before commencement of the experiment.

The healthy rats were fasted overnight and diabetes was then induced with a single intraperitoneal injection of a freshly prepared streptozotocin (STZ in 0.1 M sodium citrate buffer pH 4.5, 60 mg/kg body weight, b.w). The diabetic rats (with fasting blood glucose level > 11 mmol/L after 5 days of STZ injection) were used for the study. 

### 3.4. Antihyperglycemic Study in Vivo

The antihyperglycemic activity of the triterpene (**RA-3**) was evaluated in STZ-induced diabetes in rats [22]. The diabetic animals were randomly divided into three groups (group III, IV and V) of five rats per group. Groups I and II served as non-diabetic control groups. The test compound (**RA-3**) and metformin were dissolved in 2% Tween 20 to a final concentration of 100 mg/kg b.w for use in the study. All the animals were orally administered once a day with the drugs as shown below.

Group I: non-diabetic rats, received 2% Tween 20 (carrier solvent)
Group II: non-diabetic rats, received **RA-3** in 2% Tween 20 (100 mg/kg b.w)
Group III: diabetic rats, received 2% Tween 20
Group IV: diabetic rats, received **RA-3** at 100 mg/kg b.w
Group V: diabetic rats, received metformin at 100 mg/kg b.w

The animals were orally administered with a single dose of the drugs daily for 14 days. Blood glucose levels were measured at seven days interval (0, 7 and 14). At the end of the 14 days, the animals were fasted for 12 h and an oral glucose tolerance test was performed.

#### 3.4.1. Oral Glucose Tolerance Test

The animals received an oral glucose load of 2 g/kg b.w. At 0, 30, 60, 120 and 180 min intervals, blood was withdrawn from the rat’s tail tip and the blood glucose level was measured using glucometer (Accutrend, Roche Products, Johannesburg, South Africa). At the end of the experiment, the animals were euthanized by anesthesia. Blood and livers were immediately collected and used for biochemical estimation assays.

#### 3.4.2. Biochemical Analysis

Biochemical estimation of antioxidant status, some glycolytic enzymes and glycogen content were conducted on the serum and liver, respectively. The liver was homogenized (1:10 *w*/*v*) in a cold phosphate buffer (0.1 M, pH 7.4) containing 2 mmol/L EDTA and centrifuged at 1000× *g* for 10 min. The supernatant was collected and used to estimate hexokinase, glucokinase, and glucose-6-phosphate activity.

#### 3.4.3. Antioxidant Status

Commercial activity assay kits (Sigma-Aldrich) were used to estimate antioxidant status, antioxidant enzymes (catalase, CAT; superoxide dismutase, SOD) activities, glutathione (GSH) and malondialdehyde (MDA) content.

#### 3.4.4. Hexokinase and Glucokinase Activity 

Hexokinase (HK) and glucokinase (GK) activity were quantified using a spectrophotometric method described by Panserat *et al*. [23] with some modification. Briefly, the reaction mixture consisted of supernatant (25 µL), 50 µL phosphate buffer (0.1 M, pH 7.4, 2 mmol/L EDTA), 10 mM NADP^+^ (25 µL), 15 mM ATP (20 µL), 100 mM magnesium chloride (25 µL) and 100 µL of 100 mM or 5 mM glucose. The reaction was initiated by addition of 20 µL of glucose-6-phosphate dehydrogenase (5 units) and incubated for 5 min at room temperature. The enzyme (HK) activity was taken as the amount of NADPH formed and this was measured at 340 nm for 3 min at 30 s interval. GK activity was estimated by finding the difference between the rate of NADPH formation in presence of 5 mM and 100 mM glucose. Enzyme activity was calculated using the formula:

$$\text{Units}/\text{mL enzyme }=\frac{\left(\text{ΔA 340 nm/min Test}-\text{ ΔA340 nm/min Blank}\right)\;\left(\text{TV}\right)\left(\text{DF}\right)}{\left(6.22\right)\left(\text{EV}\right)}$$

where, TV = total volume; DF = dilution factor; EV = enzyme volume; 6.22 = extinction coefficient (mM) of β-NADPH.

#### 3.4.5. Glucose-6-phosphatase Activity Assay

Glucose-6-phosphatase (G-6-Pase) activity was assayed according to the method of Swanson [24] with some modifications. The enzyme activity was determined by estimation of inorganic phosphate (P_i_) liberated from glucose-6-phosphate (G6P). Supernatant was used as the enzyme source. The reaction mixture contained 0.1 mL of 0.1 M glucose-6-phosphate solution, 0.3 mL of 0.5 M maleic buffer (pH 6.5) and 0.05 mL of homogenate and was incubated at 37 °C for 15 min. Trichloroacetic acid (10%) was then added to terminate the reaction. The liberated P_i_ was determined with ammonium molybdate. Ascorbic acid was used as a reducing agent. Absorbance of the phosphor-molybdous complex (blue) was read at 700 nm and the enzyme activity was expressed as mg of P_i_ liberated per time unit.

#### 3.4.6. Liver Glycogen Content

The liver glycogen content was determined according to the method of Ong and Khoo [25]. Briefly, 1 g of a frozen liver was thawed and homogenized in 5 mL of ice-cold potassium hydroxide solution (30%) and boiled at 100 °C for 30 min. Ethanol (95%, Merck) was used to precipitate glycogen, pellet was washed and resolubilised in distilled water. The solubilized pellet was then treated with anthrone reagent and absorbance was read at 630 nm. The amount of glycogen in the tissue sample was expressed in mg/g tissue.

### 3.5. Data Analysis

Unless stated otherwise, all experiments were replicated at least three times. Data were reported as mean ± S.E.M. One way analysis of variance (ANOVA), followed by Dunnett’s post hoc test (GraphPad Prism version 5.03) were used to determine statistical differences. The values were considered statistically significant where *p* < 0.05.

## 4. Conclusions

The results obtained indicate anti-hyperglycemic activity of the lanosteryl triterpene (methyl-3β-hydroxylanosta-9,24-dien-21-oate, **RA-3**) from *P. longifolia.* It is apparent that the compound exerts its hypoglycaemic effect through enhancement of cellular glucose uptake and utilization, along with reduction of oxidative stress. Lack of cytotoxicity of **RA-3** which has recently been demonstrated in myocytes and adipocytes [13] further encourages the potential use of this compound in the development of a new safe pharmacologically active antidiabetic drug. Bioavailability and pancreatic β-cells’ protective effect of the triterpene are recommended for future studies.

## References

[1] Shaw J.E., Sicree R.A., Zimmet P.Z. (2010). Global estimates of the prevalence of diabetes for 2010 and 2030. Diabetes Res. Clin. Pract..

[2] Stolar M.W., Hoogwerf B.J., Gorshow S.M., Boyle P.J., Wales D. (2008). Managing type 2 diabetes: Going beyond glycemic control. Manag. Care Pharm..

[3] Ortiz-Andrade R.R., Garcίa-Jiménez S., Castillo-España P., Ramίrez-Avilla G., Villalobos-Milona R., Estrada-Soto S. (2007). Alpha glucosidase inhibitory activity of the methanolic extract from *Tournefortia hartwegiana*: An anti-hyperglycemic agent. J. Ethnopharmacol..

[4] Gutierrez R.M.P. (2013). Evaluation of the hypoglycemic and hypolipidemic effects of triterpenoids from *Prosthechea michuacana* in STZ-induced type 2 diabetes in mice. Pharmacologia.

[5] Ramachandran S., Rajasekaran A., Manisenthilkumar K.T. (2012). Investigation of hypoglycemic, hypolipidemic and antioxidant activities of aqueous extract of *Terminalia paniculata* bark in diabetic rats. Asian Pac. J. Trop. Biomed..

[6] Hung H., Qian K., Morris-Natschke S.L., Hsu C., Lee K. (2012). Recent discovery of plantderived anti-diabetic natural products. Nat. Prod. Rep..

[7] Nankar R.P., Doble M. (2013). Non-peptidyl insulin mimetics as a potential antidiabetic agent. Drug Discov. Today.

[8] Hou W., Li Y., Zhang Q., Wei X., Peng A., Chen L., Wei Y. (2009). Triterpene acids isolated from *Lagerstroemia speciosa* leaves as α-glucosidase inhibitors. Phytother. Res..

[9] Santos F.A., Frota J.T., Arruda B.R., de Melo T.S., da Silva A.A., Brito G.A., Chaves M.H., Rao V.S. (2012). Antihyperglycemic and hypolipidemic effects of α, β-amyrin, a triterpenoid mixture from *Protium heptaphyllum* in mice. Lipids Health Dis..

[10] Lee M.S., Phuong T.T. (2010). Stimulation of glucose uptake by triterpenoids from *Weigela subsessilis*. Phytother. Res..

[11] Alqahtani A., Hamid K., Kam A., Wong K.H., Abdelhak Z., Razmovski-Naumovski V., Chan K., Li K.M., Groundwater P.W., Li G.Q. (2013). The pentacyclic triterpenoids in herbal medicines and their pharmacological activities in diabetes and diabetic complications. Curr. Med. Chem..

[12] Machaba K.E., Cobongela S.Z.Z., Mosa R.A., Lawal A.O., Djarova T.G., Opoku A.R. (2014). *In vivo* anti-hyperlipidemic activity of the triterpene from the stem bark of *Protorhus longifolia* (Benrh). Engl. Lipids Health Dis..

[13] Mosa R.A., Naidoo J.J., Nkomo F.S., Mazibuko S.E., Muller C.J.F., Opoku A.R. (2014). *In vitro* anti-hyperlipidemic potential of triterpenes from stem bark of *Protorhus longifolia*. Planta Med..

[14] Khathi A., Serumula M.R., Myburg R.B., Van Heerden F.R., Musabayane C.T. (2013). Effects of *Syzygium aromaticum*-derived triterpenes on postprandial blood glucose in streptozotocin-induced diabetic rats following carbohydrate challenge. PLoS ONE.

[15] Jang S.M., Kim M.J., Choi M.S., Kwon E.Y., Lee M.K. (2010). Inhibitory effects of ursolic acid on hepatic polyol pathway and glucose production in streptozotocin-induced diabetic mice. Metab. Clin. Exp..

[16] Ramachandran V., Saravanan R. (2013). Efficacy of asiatic acid, a pentacyclic triterpene on attenuating the key enzymes activities of carbohydrate metabolism in streptozotocin-induced diabetic rats. Phytomedicine.

[17] Abd El-Baky A.E. (2011). Quercetin protective action on oxidative stress, sorbitol, insulin resistance and β-cells function in experimental diabetic rats. Int. J. Pharm. Stud. Res..

[18] Ghosh T., Maity T.K., Singh J. (2011). Antihyperglycemic Activity of Bacosine, a Triterpene from Bacopa monnieri, in Alloxan-Induced Diabetic Rats. Planta Med..

[19] Castellano J.M., Guinda A., Delgado T., Rada M., Cayuela J.A. (2013). Biochemical basis of the antidiabetic activity of oleanolic acid and related pentacyclic triterpenes. Diabetes.

[20] Vinoth-kumar V., Ramesh N., Bricey A.A., Selvi V.V.T. (2010). Evaluation of lipid peroxidation and antioxidants activity of metformin in high fructose fed diet induced type II diabetic rats. Int. J. Pharm. Technol..

[21] Sani M.F., Kouhsari S.M., Moradabadi L. (2012). Effects of three medicinal plants extracts in experimental diabetes: Antioxidant enzymes activities and plasma lipids profiles in comparison with metformin. Iran. J. Pharm. Res..

[22] Kumar S., Kumar V., Prakash O.M. (2012). Antidiabetic and hypolipidemic activities of *Kigelia pinnata* flowers extract in streptozotocin induced diabetic rats. Asian Pac. J. Trop. Biomed..

[23] Panserat S., Capilla E., Gutierrez J., Frappart P.O., Vachot C., Plagnes-Juana E., Aguirrea P., Brequea J., Kaushika S. (2001). Glucokinase is highly induced and glucose-6-phosphatase poorly repressed in liver of rainbow trout (*Oncorhynchus mykiss*) by a single meal with glucose. Comp. Biochem. Physiol. Part B.

[24] Swanson M.A., Colowick S.P., Kaplan N.O. (1995). Glucose-6-phosphatase from Liver. Methods in Enzymology.

[25] Ong K.C., Khoo H.E. (2000). Effects of myricetin on glycemia and glycogen metabolism in diabetic rats. Life Sci..

